# A Glimpse to Background and Characteristics of Major Molecular Biological Networks

**DOI:** 10.1155/2015/540297

**Published:** 2015-09-30

**Authors:** Md. Altaf-Ul-Amin, Tetsuo Katsuragi, Tetsuo Sato, Shigehiko Kanaya

**Affiliations:** ^1^Graduate School of Information Science, Nara Institute of Science and Technology, 8916-5 Takayama-cho, Ikoma-shi, Nara 630-0192, Japan; ^2^Department of Computer Science and Engineering, Toyohashi University of Technology, 1-1 Hibarigaoka, Tempaku-cho, Toyohashi-shi, Aichi 441-8580, Japan

## Abstract

Recently, biology has become a data intensive science because of huge data sets produced by high throughput molecular biological experiments in diverse areas including the fields of genomics, transcriptomics, proteomics, and metabolomics. These huge datasets have paved the way for system-level analysis of the processes and subprocesses of the cell. For system-level understanding, initially the elements of a system are connected based on their mutual relations and a network is formed. Among omics researchers, construction and analysis of biological networks have become highly popular. In this review, we briefly discuss both the biological background and topological properties of major types of omics networks to facilitate a comprehensive understanding and to conceptualize the foundation of network biology.

## 1. Introduction

In molecular biology, the list of components at the genome, transcriptome, proteome, and metabolome levels is gradually becoming complete and well-known to scientists. However, it is not holistically known how these components interact with each other to grow, maintain, and reproduce life at different phases, in different environments or with different challenging conditions. In cells, many concurrent and sequential tasks are performed based on complex signaling and regulation. Different omics molecules are elements of a cell. Due to the existence of unicellular organisms, cells are, in some sense, considered a basic unit of life.

For system-level understanding, initially the elements are connected based on their mutual relations and a network is formed on this basis [1]. Networks at the molecular level are constructed to understand and explain the cell as a system. In multicellular organisms, cells that constitute tissues and organs in turn are organized and arranged to make an organism. Like intracellular signaling, there is also intercellular signaling. A whole organism can be viewed as a network of cells or as a network of organs at a higher level. An ecosystem, in turn, is made of many organisms and depends on species-species relations. A network of species can be constructed and utilized to understand and analyze ecosystems. Furthermore, over time, an organism can evolve and to explain such evolution phylogenetic networks, mostly trees, have been constructed involving present and past organisms.

In recent years, substantial research has been conducted on networks ranging from social and biological networks up to the Internet, aiming to decipher mechanisms of how these networks grow and evolve and what global properties they develop in the long run. Global network properties such as average path length, clustering coefficient, and degree distribution reflect useful information about the nature of these networks such as network robustness and the existence of hub nodes or clusters [1, 2]. Network theory also allows calculation of different centrality measures for network elements, revealing globally important network information in different contexts [3–5]. Centrality measures can identify various things including determination of nodes that disseminate information rapidly in the network or which nodes to consider for blocking the spread of something in the network.

It is increasingly commonly recognized that complex systems cannot be described by separately studying individual elements. Analysis and understanding of the behavior of such systems start with determination of the global topological properties of the corresponding network. Cellular molecules mainly consist of DNA, RNA, proteins, and metabolites, which are the key drivers of cellular mechanisms. The actions and interactions of such molecules control various functions of the cells. In the present review, we focus on molecular biological networks. In such networks, the nodes are usually cellular molecules such as genes, proteins, or metabolites, while the edges represent biological relationships, for example, physical interactions, regulations such as activation and inhibition of gene expression, or reactions such as substrate product association. Networks in systems biology, can be constructed in different contexts and sizes to support to system-level analyses of cellular processes, subprocesses, or higher-level biological phenomenon.

The concept of networks and network-based methods finds many applications in systems biology [1, 6]. Relational networks of genes derived from gene expression data can be used to develop novel biological hypotheses about subgenome level interactions and mechanisms such as signaling and regulation to guide new experimental designs aimed at testing such hypotheses [7]. Biological networks can be utilized to identify biomarkers for disease diagnosis. Even a subnetwork could be used to identify biomarkers for diagnostic, predictive, or prognostic purposes [8–11]. Protein network and mRNA profiles can be integrated to identify subnetwork biomarkers, that is, highly connected genes of a subnetwork that could be the marker of a disease state. There are several network-based approaches for identifying disease genes and protein interaction subnetworks which are disease signatures [12–14].

There is growing evidence that a network approach is needed for successful development of medications for complicated diseases [15]. Complicated noncommunicable diseases such as cancer, Alzheimer's disease, mental disorders, and heart diseases are caused by multiple molecular abnormalities. The drug discovery process for these diseases requires targeting entire molecular pathways of various cellular omics networks rather than single molecules.

Recently, biological networks, for example, protein-protein interaction (PPI) networks and gene expression networks, have found widespread application in drug target detection [16–19]. A system-level approach to function prediction of unknown omics molecules can be performed by constructing a network of such molecules and by analyzing the clusters in the network based on the “guilt by association” philosophy [1].

Already, omics networks have become an indispensable part of understanding biology and medicine, and they will be increasingly important in the future. In this paper, we discuss the background and characteristics of some basic types of molecular biological networks. This information provides a useful foundation for understanding the concepts of biological systems.

The rest of this paper is organized in several sections.  Section 2 describes the gene regulatory networks, including the biological mechanism, regulatory relations, and topological properties.  Section 3 discusses the protein-protein interaction networks, defining these networks, how they are detected, and their properties.  Section 4 describes the biology and properties of metabolic pathways.  Section 5 looks at signal transduction and signaling networks.  Section 6 then examines the growing number of databases related to omics networks. Finally, Section 7 draws our conclusions from this review of the background and characteristics of major molecular biological networks.

## 2. Gene Regulatory Networks

In this section we briefly describe the biological mechanism of gene regulation, determination methods of regulatory relations between genes, and the topological properties of gene regulatory networks.

### 2.1. The Biology of Gene Regulation

The main objective of gene regulation is to regulate the production of proteins, which are directly associated with development, maintenance, and survival of organisms. The process of producing proteins has several steps, from DNA transcription to mRNA through translation to proteins, all of which are controlled by the gene regulation system. Chromosomes contain DNA, a double helix of nucleotide sequences, which contains codes for many proteins separated by noncoding regions. Generally, the code for a single protein on the DNA is called a gene. To produce a protein, first the DNA corresponding to a gene is transcribed to an mRNA by a molecular machine called RNA polymerase. An mRNA is a single-stranded nucleotide sequence that usually contains the code of a single protein or sometimes more proteins. This process of producing an mRNA from DNA is known as transcription, while generation of mRNAs of a gene is called expression of a gene. From there, another molecular machine called a ribosome extracts the information from the mRNA and produces proteins. This process is known as translation. The total process of information flow and protein production from DNA through mRNA to protein is generally known as the central dogma of molecular biology. However, the gene regulation system controls this process, determining which protein is produced, how much, where, and when. Gene regulation requires very complex signal control for proper development, maintenance, and survival of an organism. While all of the mechanisms and information about the regulatory systems of all genes are not yet known, it is clear that deciphering the gene regulation system is important for treating complex diseases and genetic engineering.

A key part of the gene regulation system is what is known as transcription factors (TFs). As described above, the process of protein production starts with transcription of the corresponding gene. Therefore, major mechanisms of gene regulation are based on the interaction of TFs and other regulators such as microRNAs (miRNAs) at the transcription level. TFs are special types of proteins that have DNA binding domains that can bind at specific sites of DNA defined by particular sequences of certain length. For example, a yeast TF, GAL4, is a chain of 881-amino acids with a Zn-Cys binuclear cluster-type DNA-binding domain [20]. The nuclear protein GAL4 is a positive regulator of gene expression for the galactose-induced genes such as GAL1, GAL2, GAL7, GAL10, and MEL1. These genes encode enzymes that convert galactose to glucose. GAL4 recognizes a 17-base-pair long sequence in the upstream activating sequence (uas-g) of these genes, (5′-cggrnnrcynyncnccg-3′) CGG-N_11_-CCG [21], where r stands for Purine (A or G), y for Pyrimidine (C or T), and n is any nucleotide.

Regulation of gene expression at the transcription level is a fundamental process that is evolutionarily conserved in all cellular systems [22]. In this mechanism, the TFs bind at specific sites in the promoter region of a gene using their DNA binding domain and thus affect the expression of the target gene (TG). The promoter is the upstream region of the transcription start site of a gene, which is composed of a short core promoter [23] and nearby regulatory elements. Also there are distal regulatory elements, which can be enhancers, silencers, insulators, or locus control regions (LCR) [24]. Despite extensive studies, we still have limited understanding of the mechanisms of distal regulatory elements [25]. The specific site where a TF physically binds is called a cis-regulatory motif.

A TF can work as an activator, a repressor, or as a dual regulator. An activator increases the expression of the TG by enhancing the activity of the RNA polymerase at the promoter. In the context of prokaryotic transcription, a TF is known to bind upstream of the transcription start site and often upstream of the −35 promoter element in case of activation. For repression, a TF usually binds the DNA to prevent RNA polymerase from initiating transcription. For repression, a TF usually binds downstream of the transcription site, causing DNA looping or, by binding between −35 and −10 elements of the promoter region, blocks RNA polymerase from binding to the DNA and initiating transcription [26, 27]. Eukaryotic promoters are of various types and are often difficult to characterize. However, recent studies show that they are divided into more than ten classes [28].

Between prokaryotes and eukaryotes, the process of transcription is somewhat different. Within the cell of a prokaryote, the nucleoid is an irregularly shaped region that contains all or most of the genetic material [29]. In contrast, in a eukaryotic cell, the nucleus is surrounded by a nuclear membrane. In prokaryotic organisms, the genome is generally a circular, double-stranded piece of DNA. Such a DNA is called a genophore, commonly referred to as a prokaryotic chromosome. In the context of chromatin, this DNA is different from that of a eukaryote. In a eukaryotic cell, chromatin is the combination of DNA and proteins that make up the contents of the nucleus. The primary protein components of chromatin are histones that compact the DNA into a smaller volume to fit in the cell and prevent DNA damage. Prokaryotes do not have typical histones, but they do have histone-like proteins that package DNA.

In prokaryotes, the absence of a nucleus facilitates transcription and translation on the same site. Prokaryotes also have known operons, that is, groups of adjacent genes that are transcribed as the same messenger RNA but translated separately. The control of transcription in prokaryotes primarily occurs at the DNA sequence level by using cis-regulatory elements.

The process of transcriptional regulation in eukaryotes is highly complicated and estimated to be coordinated and controlled at several steps, including transcription initiation and elongation and mRNA processing, transport, translation, and stability [24]. Most regulation, however, is believed to occur at the level of transcription initiation by the RNA polymerase. Many biological events, including chromatin condensation, DNA methylation, alternate splicing of RNA, mRNA stability, translational control, protein degradation, and regulation by noncoding RNA, can be regarded as mechanisms of gene regulation [30]. The noncoding RNAs called miRNA are important regulators of gene expression. They are conserved across species, expressed across cell types, and active against a large proportion of the transcriptome. miRNAs are ~22-nucleotide RNAs that posttranscriptionally repress gene expression by base pairing to mRNAs [31].

A number of research studies have examined transcription regulation based on relations between TFs and targeted genes. A set of such relations is called a transcriptional regulatory network (TRN), which may be considered a type of gene regulatory network (GRN). Any comprehensive characterization of GRNs must include TF-DNA-binding specificities as well as higher-order modes of regulation such as protein modification and protein-protein interaction [32]. The concept of a GRN is somewhat broader than that of a TRN and a comprehensive GRN may include relations other than transcriptional regulations involving other molecules such as miRNA and even metabolites. Genes may have various types of relations between them, for example, transcriptional regulatory relations, or they may be concerned with the same protein complex or metabolic/signaling pathways. Obviously, gene expression data should contain some clues to such relations [33]. A GRN, then, is defined as a network that has been inferred from gene expression data by the application of a statistical inference method [7, 34]. Since gene expression data often quantifies the abundance of mRNAs, a GRN provides information about general interactions, other gene-gene interactions, and potential protein interactions such as in a complex [34].

### 2.2. How Regulatory Relations Are Determined

The transcriptional relation between a TF and a TG is a kind of regulatory relation. Such relations are determined by small-scale or high-throughput methods to define the protein-DNA interactions. Various methods such as ChIP-chip and ChIP-seq can directly infer* in vivo* binding of TF to TGs [35, 36]. Both experimental and computational methods are currently used to discover and characterize the TF-TG binding interactions. Marcel and Sebastian reviewed the experimental strategies for studying TF-TG binding specificities [37].

Another approach to assess transcriptional regulatory relations is to determine differentially expressed genes upon overexpression and deletion of TFs. Regulatory relations between genes can be modeled by analyzing time series or specific perturbation-based expression data of a comprehensive set of genes. GRN modeling is often performed based on Boolean or Bayesian networks or differential or difference equations or by determining expression profile similarities between genes based on some measure such as correlation, Euclidean distance, or mutual information [38–41]. Reverse engineering gene networks based on gene expression data using singular value decomposition and robust regression have also been proposed [42]. However, it is still a challenge to reconstruct underlying regulatory systems from noisy experimental data, due to stochastic biological dynamics and nonlinear interactions. Emmert-Streib et al. reviewed the methods for inferring gene regulatory networks from observational gene expression data in detail [34, 43].

### 2.3. Properties of Regulatory Networks

The combination of all regulatory relations between TFs and TGs of a species can be regarded as a static network. In general, one gene may be regulated by more than one TF, and one TF may regulate more than one gene. The TFs themselves may be regulated by the same or other TFs. One of the global topological properties of such a static network is its degree distribution. The degree distribution is the probability distribution function *p*(*k*), which is a function of degree *k*. The function *p*(*k*) shows the probability that the degree of a randomly selected node in the network is *k* [44]. Usually, in the case of biological networks, the degree distributions are represented as frequency distributions instead of probability distributions, and corresponding to both the approaches the shape of the distribution remains the same. Degree distributions of TRNs have been analyzed for several species. Overall, the connectivity follows power law (*p*(*k*) ~ *k* ^−*γ*^) with *γ* ≈ 2 in the case of* E. coli* and* S. cerevisiae* [45, 46]. Networks for which degree distribution follows power law are highly nonuniform; that is, most of the nodes have only a few links, with a few nodes that have many links. TRNs are directed networks because the edges are directed from TFs to TGs. For such networks, indegree and outdegree distributions can be estimated separately. In a separate study, it was shown that indegree distribution follows exponential law (*p*(*k*) ~ *Ae* ^−*αk*^) while outdegree distribution follows power law in the case of a typical* S. cerevisiae *regulatory network [45]. Exponential indegree distribution implies that a similar number of TFs regulate most TGs. However, the power law outdegree distribution implies that there are hub TFs in the network which regulate a disproportionately large number of TGs. Such hub TFs are usually called global regulators [47]. As certain TFs regulate other TFs, it is possible to discover a hierarchical structure in a TRN. Indeed, a number of studies have determined a hierarchical structure in a TRN using both top-down and bottom-up approaches [48, 49].

**Figure 1:**
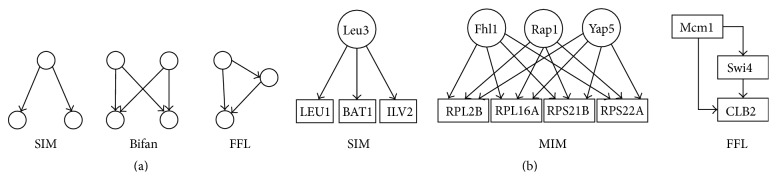
(a) Well-known motifs in transcriptional regulatory networks and (b) some real examples of SIM, MIM, and FFL.

Other studies have determined the occurrence of certain motifs in TRNs.  Figure 1(a) shows the structure of common network motifs, namely, a feed forward loop (FFL), bifan, and single input motif (SIM). The bifan is a special case of the more general type multiple input motif (MIM).  Figure 1(b) shows real examples of SIM, MIM, and FFL [50]. The FFLs can be of two types: coherent and incoherent depending on the match and mismatch, respectively, of the regulatory effects via the direct and feed forward paths [51]. In another work studying the dynamic structure of the TRN of yeast, five subnetworks were generated based on the static TRN, two of them related to cell cycle and sporulation (endogenous conditions), and the other three related to dioxic shift, DNA damage, and stress response (exogenous conditions) [52]. This study showed that FFLs are overrepresented in the networks related to endogenous conditions, whereas single input motifs are overrepresented in the networks related to exogenous conditions.

## 3. Protein-Protein Interaction (PPI) Networks

Here we discuss PPI network concepts, how PPIs are determined, and the properties of PPI networks.

### 3.1. What Is a PPI Network?

In cells, thousands of different types of proteins act as enzymes, catalysts to chemical reactions of the metabolism, components of cellular machinery such as ribosomes, regulators of gene expression, and so on. Some proteins play specific roles in special cellular compartments, whereas others move from one compartment to another carrying mass or information.

**Figure 2:**
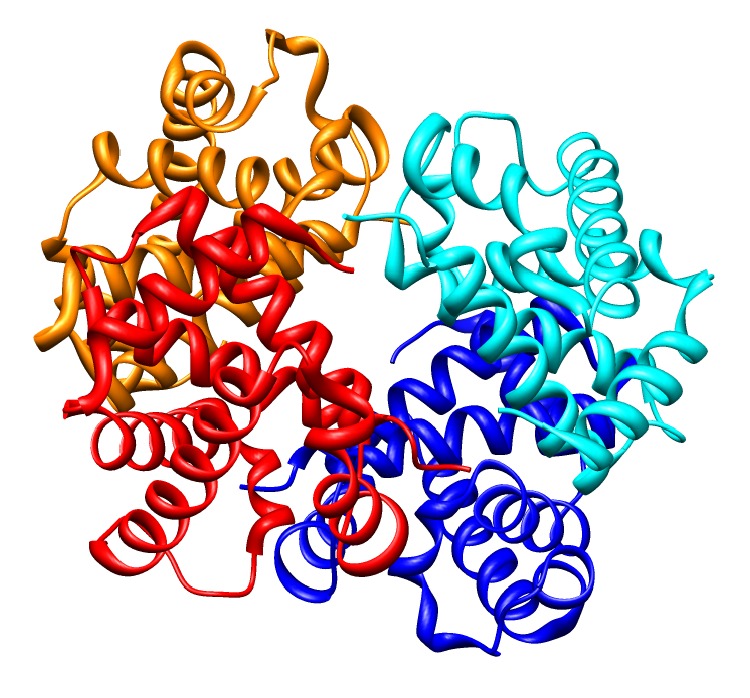
Cartoon sketch of hemoglobin tetramer (PDB ID: 1GZX).

Usually, more than one protein physically interacts or binds with other proteins to form a complex performing certain biological tasks. For example, in adult humans, the most common hemoglobin type is a tetramer (which contains 4 subunit proteins), consisting of two *α* and two *β* subunits noncovalently bound, each made up of 141 and 146 amino acid residues, respectively. The subunits are structurally similar and about the same size. In human infants, the hemoglobin molecule is made up of 2 *α* chains and 2 *γ* chains. The gamma chains are gradually replaced by *β* chains as the infant grows. Salt bridges, hydrogen bonds, and the hydrophobic effect keep the four polypeptide chains together. The hemoglobin tetramer is a good example of physical interaction between proteins to form a protein complex.  Figure 2 shows a typical cartoon image of a hemoglobin tetramer. Numerous PPIs thus construct useful complexes to perform biologically important tasks. A PPI network usually refers to a network made of proteins as nodes, with known or predicted interactions between them as edges. Usually, for global analysis, all known and predicted interactions in an organism are used to construct a large PPI network.

### 3.2. Detection of Protein Interactions

There are various ways to detect protein interactions. A comprehensive list of the different experimental procedures can be found in scientific literatures [79, 80]. The two most popular high-throughput methods are the yeast two-hybrid system (Y2H) [81] and affinity purification coupled to mass-spectrometry (AP-MS) [82]. Below we discuss some details about the Y2H system.

As an example, we discuss Y2H method in the context of the GAL4 protein. GAL4 is a global TF that activates galactose metabolic pathways. It has a DNA binding domain (BD) that binds to the specific sequence upstream of the GAL4 regulated genes and an activating domain (AD) which binds to other proteins to activate the transcription. Both domains are small parts of GAL4 proteins and are capable of functioning independently but they need to be in close proximity. If these two domains are expressed as separate polypeptide chains in the same cell, they are not in close proximity and thus they fail to activate transcription. It is therefore reasonable to hypothesize that if BD is fused to protein P1 and AD is fused to protein P2, with both fusions coexpressed in the same cell so that the transcription of GAL4 regulated genes can be activated; then we conclude that P1 and P2 physically interacted to bring BD and AD into close proximity. Y2H systems exploit this idea to determine interactions between two unknown proteins. A “bait” is constructed by fusing a protein, such as P1 to BD, and a “prey” is constructed by fusing another protein, such as P2 to AD, and both fusions are coexpressed in the same reporter cell. Then the expression level of GAL4 regulated genes is measured to determine whether P1 and P2 interact.

Fields and Song pioneered Y2H in 1989 [83]. Since then, the same principle has been adapted to describe many alternative methods, including some that detect protein-DNA interactions [84] or those that detect DNA-DNA interactions and use* Escherichia coli* instead of yeast [85]. Large-scale two-hybrid studies have also been used to study interactions in yeast [81],* Caenorhabditis elegans* [86],* Drosophila melanogaster* [87], and humans [88].

The other popular method for detecting PPI is AP-MS. The details of this method can be found in [82, 89, 90]. Studies such as [91, 92] utilized it, where each work identified roughly 300 protein complexes in yeast.

### 3.3. Insights into Protein Interaction Networks

Other than degree distribution, two other global topological properties of a network are average path length and clustering coefficient [44]. A path between two nodes in a graph is a sequence of edges, starting from one node and ending at the other. The distance between two nodes is the length of the shortest path between them. In a graph consisting of *N* nodes, there are $\left(\begin{array}{c} N\\ 2 \end{array}\right)=N(N-1)/2$ distinct node pairs, and the average path length of the graph is defined as the average distance between all possible node pairs. The clustering coefficient of a node is the ratio of the actual number of edges and the maximum possible number of edges among its neighbors. The clustering coefficient of a graph, then, is the average of the clustering coefficients of all its nodes.

It has been shown that for random networks both the average path length and the clustering coefficient are low, while for PPI networks the average path length is low, but the clustering coefficient is high, identifying such networks as the “small-world” type [44]. The high clustering coefficient indicates that there are high-density modules in the networks. A number of algorithms have been developed to identify high-density modules in PPI networks [93–96]. Such modules show relevance to the known protein complexes.

The degree distribution of PPI networks is reported to be of power-law type (*p*(*k*) ~ *k* ^−*γ*^) [44]. The power-law degree distribution indicates that the structure of the PPI networks is of “scale-free” type, which means there are a few high-degree hub nodes and many low-degree peripheral nodes. It has been reported that many of the hub nodes of PPI networks are essential, evolutionarily conserved proteins serving central roles in cellular processes [97]. The nodes of a network can be ranked based on their degree and also based on other centrality measures such as betweenness [98] or eigenvector centrality [99]. The proteins in the PPI networks for which such centrality measures are high are also more likely to be essential proteins. A PPI network of yeast was shown to be a combination of high-density and star-like modules [93].

**Figure 3:**
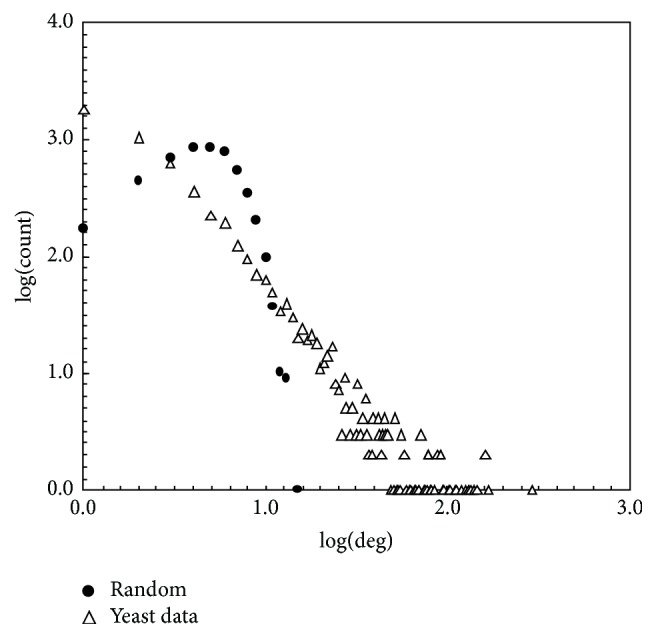
The degree distribution of a yeast PPI network and that of a random network of the same size.

Figure 3 shows the degree distribution of a PPI network and a random network of equal size. The PPI network of* S. cerevisiae* consists of 12487 unique binary interactions involving 4648 proteins collected from the Munich Information Center for Protein Sequences (MIPS) database [100]. Notice that the degree distribution of the PPI network is of power-law type while that of the random network follows Poisson's distribution (*p*(*k*) = *e* ^−*λ*^ *λ* ^*k*^/*k*!) [101].

## 4. Metabolic Pathways

In this section, we discuss the biological basics of metabolic pathways and their properties.

### 4.1. Biological Basics of Metabolic Pathways

**Figure 4:**
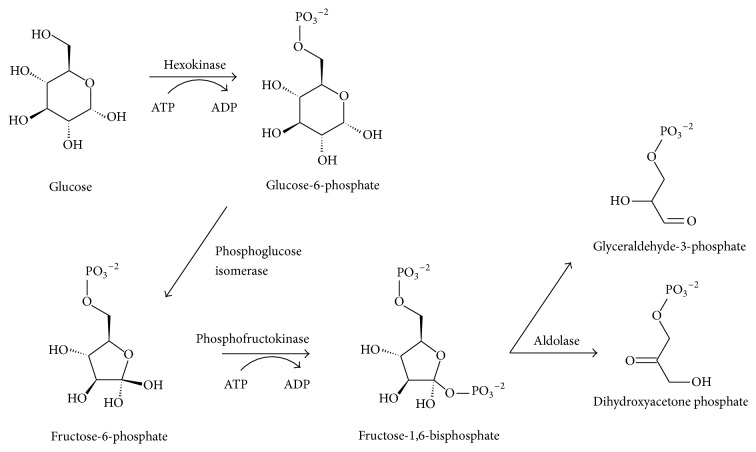
The first stage of glycolysis.

Living cells generate energy and produce building material for cell components and replenishing enzymes by the process of metabolism. All organisms live and grow by receiving food or nutrients from the environment and assimilating those chemicals. The foods are processed through thousands of reactions. In cells, chemical reactions take place constantly, breaking and making chemical molecules and transferring ions and electrons. These reactions are typical of metabolic pathways. As an example, the first stage of glycolysis pathway is shown in Figure 4. The glycolysis pathway is very primitive in terms of evolution and is common to essentially all living organisms. Metabolites can therefore be considered as the preliminary level molecules generated from food intakes which are gradually transformed into building blocks for producing proteins, RNAs, and DNAs, along with other useful matter and energy for creating and maintaining cells and life.

Metabolic reactions follow the laws of physics and chemistry, so modeling metabolic reactions requires considering many physicochemical constraints [102]. Considering the balance of inflow and outflow of every chemical reaction within the entire metabolic network, we can estimate reaction flow under a steady state and predict optimal performance for bioproduction [103]. However, it is still difficult to model dynamic behavior of the whole metabolic network, since kinetic parameters and the regulatory interaction of enzymes are not fully determined. Actually, to respond to external perturbations and internal needs, metabolic pathways must be efficiently regulated, so they are linked to signaling networks. Metabolic imbalance causes many severe human diseases such as diabetes, cancer, cardiovascular problems, obesity, gout, and tyrosinemia.

Metabolism is a general term for two kinds of reactions, catabolic and anabolic reactions. Catabolic reactions refer to chemical reactions that break more complex organic molecules into simpler substances. They usually release energy that drives chemical reactions. In these reactions, large molecules such as polysaccharides, lipids, nucleic acids, and proteins are broken down into smaller units such as monosaccharides, fatty acids, nucleotides, and amino acids. The energy from catabolic reactions is used to drive anabolic reactions. Anabolic reactions refer to chemical reactions in which simpler substances are combined to form more complex molecules. These reactions usually require energy to build new molecules and/or store energy. The energy for chemical reactions is stored in adenosine triphosphate (ATP).

The term metabolic network usually means a collection of metabolic reactions represented as networks, where the metabolites are the nodes, and two metabolites are connected if one of them is a substrate and the other is the product of a reaction. Genome scale reconstruction of a metabolic network involves thousands of metabolites and reactions. Metabolic reactions are catalyzed by enzymes, which in a broader sense are themselves gene products or proteins. Metabolic networks therefore contain information about both metabolites and proteins where the metabolites are nodes and the proteins/enzymes are edges. There are other ways of representing metabolic pathways, such as Bipartite graphs or Petri nets [104, 105]. A metabolic pathway can be represented as a bipartite graph by considering the metabolites as one set of nodes and the enzymes as another set of nodes. Such a representation can provide some overall preliminary information about the system.

### 4.2. Characteristics of Metabolic Pathways

Usually, large-scale metabolic pathways are represented as networks by replacing the enzymes/reactions as unidirectional/bidirectional edges and keeping the metabolites as nodes. However, to make it biologically meaningful, usually the currency metabolites are excluded from the network. There is no strict definition of the currency metabolites. However, the metabolites that are used as carriers for transferring electrons and certain functional groups such as phosphate, amino, or methyl group are often called currency metabolites. Different studies use different sets of currency metabolites for the sake of extracting meaningful results. One study showed that even when currency metabolites are included in the network, metabolic networks are scale-free networks, that is, their degree distribution follows power law [106]. The work of Ma and Zeng [107] found that, after deletion of the currency metabolites, the structure of the metabolite networks still has a scale-free structure.

Overall, metabolic networks can be regarded as small-world networks for their power-law degree distribution, high clustering coefficient, low average path length, and diameter [108]. High clustering coefficient implies the existence of high-density modules in the networks. It has been proposed that the combined properties of power-law degree distribution and high clustering coefficient indicate that modules in the networks are linked to one another in a hierarchical manner [3]. It implies physicochemical constraints and evolutionary bias in development of metabolic networks, exemplified by living organisms acquiring new reaction paths by slight modification of existing enzymes. When a network consists of many small, highly integrated modules and the modules are hierarchically organized, such a network is called a hierarchical network. The most important signature of hierarchical modularity is that the average clustering of nodes of degree *k* defined as *C*(*k*) follows the power law (*C*(*k*) ~ *k* ^−*γ*^) [44]. It has been reported that hierarchical modularity exists in metabolic networks of* E. coli* and* S. cerevisiae *[106, 109].

Topological features of metabolic networks can also be used to compare taxa from different kingdoms of life, for example, archaea, bacteria, and eukarya [106, 107, 110]. These studies show that some properties are shared by all taxa; for example, the metabolic networks show scale-free structure, but other properties are different; for example, bacteria have a shorter average path length than archaea and eukarya. Also, compared to the metabolic networks of bacteria and eukarya, those of archaea have a lower average clustering coefficient, betweenness centrality, and scale-freeness [110]. Furthermore, the organization of metabolism can be linked to the species' lifestyle and phenotype, for example, to the variability of habitat [111] and growth temperature [112]. Another study used a novel representation of metabolic networks, called a network of interacting pathways (NIP) and tried to identify the most relevant aspect of cellular organization that changes under evolutionary pressure [113]. This work focused on the transitions from prokarya to eukarya, from unicellular to multicellular eukarya, from free living to host-associated bacteria, and from anaerobic to aerobic respiration, in the context of the structure of NIPs.

## 5. Signaling Networks

In this section, we discuss the basic mechanism of signal transduction, along with the differences of signaling networks from metabolic and regulatory networks. In addition, we provide examples of signal transduction systems and the properties of signaling networks.

### 5.1. Mechanism of Signal Transduction

**Figure 5:**
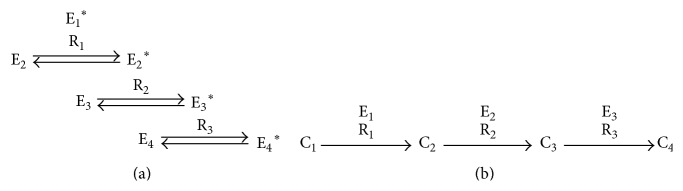
Illustration of difference between signaling and metabolic pathways.

Signaling networks are above the gene regulatory networks. Signaling networks are related to the transduction of “signals,” usually from outside to inside the cell. At the molecular level, signaling involves the same type of processes as metabolism, such as production and degradation of substances, molecular modifications (mainly phosphorylation but also methylation and acetylation), and activation or inhibition of reactions, although signaling is about changes in protein activity involving conformational changes of proteins, while metabolism is primarily about changes in small molecules. Furthermore, signaling pathways mainly serve for information processing or transfer of information, while metabolism provides mainly mass transfer [114]. To clarify the difference between the metabolic network and signal transduction network, as depicted in Figure 5, we can compare the generalized topology of a signal transduction pathway with that of a metabolic pathway.  Figure 5(a) shows that, in a signaling pathway, one active enzyme E1 modulates the activity of the another enzyme E2, which in turn modulates the activity of a third enzyme E3 without being consumed by the reaction. On the other hand, Figure 5(b) shows that, in metabolic reactions, the substrate metabolites are consumed by the reactions to produce new metabolites, and the reactions are catalyzed by the enzymes. In signal transduction pathways, the state of the enzymes toggles between on and off to propagate a signal, while, in metabolic pathways, the enzymes work as catalysts and are produced in the cell when needed. This production of enzymes may be a result of some signal propagation, which indicates that signaling networks are related to regulatory networks. But there are good reasons for treating signaling networks separately from regulatory networks [115]. Signaling networks are strongly defined by their structural layers—input, intermediate, and output—which involve crosstalk, integrated decision making, and feedforward and feedback control [116]. Thus they are different from regulatory networks, which are strongly determined by feedback loops [116]. However, all the interfaces between signaling and regulation are not known [117].

Although lipids, proteins, and metabolites are the principal components of signaling networks, further research in molecular biology may uncover additional signaling components [118], such as the discovery of the regulatory functions of miRNAs [119]. From an engineering perspective, the components of a signaling pathway can be viewed as sensors, transducers, and actuators [120]. The general sequence of steps in signal transduction are (i) binding of a ligand to a receptor, usually to an extracellular receptor embedded in the cell membrane, (ii) phosphorylation of intracellular enzymes, (iii) amplification and propagation of the signal, and (iv) consequential changes in the cellular function, for example, increase/decrease in the expression of one or more genes.

### 5.2. Examples of Signal Transduction Systems

Mitogen-activated protein kinase (MAPK) cascades are a well-known signal transduction system that is a particular part of many signaling pathways. In response to a range of stimuli, MAPKs propagate signals from the cell membrane to the nucleus. MAPK cascades are widely involved in eukaryotic signal transduction for a variety of cellular processes, including cell growth, differentiation, transformation, and apoptosis. It is worth noting that MAPKs pathways are conserved from yeast to mammal.

**Figure 6:**
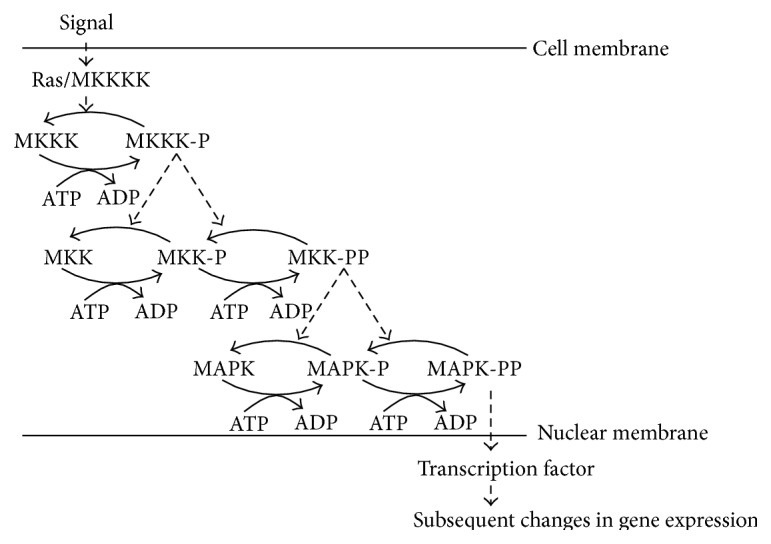
General format of the MAPK cascade.

Figure 6 shows the general format of a MAPK cascade. The signal propagates through several levels, usually 3, by phosphorylation of the MAPKs, and acts as an enzyme for the phosphorylation of the next stage MAPKs. There are several mechanisms to activate MAPKKKs by phosphorylation of a tyrosine residue. The active MAPKK kinase MAPKKK-P phosphorylates MAPK kinase MAPKK at serine and threonine residues to produce MAPKK-PP. The terminal level is the MAP kinase MAPK, and MAPKK-PP phosphorylates MAPK at two sites: conserved threonine and tyrosine residues to produce MAPK-PP which is the active state signal for the downstream. At all levels, dephosphorylation is assumed to occur continuously by phosphatases or autodephosphorylation. Some other important signal transduction mechanisms are G-protein signaling [114] and JAK-STAT pathways [114].

### 5.3. Towards Genome-Scale Signaling Networks

Like organism-wide gene regulatory networks, PPI networks, and metabolic pathways, it is also essential to construct genome-wide signal transduction networks. Signaling networks work as interfaces between the environment, the genome, and metabolism, so reconstructing genome-scale signaling networks is useful for understanding complex diseases and developing therapies [115]. Though many details of different signal transduction pathways are known, they are often fragmented, with different fragments referring to different species and cell types, making the task of constructing the large-scale signal transduction network problematic. To overcome this problem, it has been suggested that the network be constructed at the genome level instead of the species level [121]. For this purpose, it is necessary to represent the molecules by their ortholog abstractions. Despite such difficulties, a network of several thousand nodes and edges can be made by collecting information from the TRANSPATH database [122]. This network is sparse and shows scale-free properties in terms of degree distribution and small-world properties in the context of its diameter and clustering coefficient [121]. It is now possible to simultaneously measure a substantial portion of the molecular components of a cell; therefore it is time to develop and test systems-level models of cellular signaling and regulatory processes, which will facilitate gaining insights into the “thought” processes of a cell [115]. Recently, a method called CCELL was proposed, for cell-scale signaling network inference over a predefined timescale using time series immunoprecipitation data based on Bayesian compressive sensing [123].

## 6. Omics Network-Related Databases

By facilitating organized curation and search options for data, currently databases have become an important part of systems biology and big data biology. In recent years, molecular biological data in different omics fields including genomics, transcriptomics, proteomics, and metabolomics have drastically expanded both in quantity and diversity. Different biological databases focus on different aspects of molecular biology, and a number of them can be directly or indirectly linked to biological networks. The major objectives of developing these databases are curation of data and allowing analysis of the data by providing useful analytical software tools. Curation includes storage, retrieval, dissemination, filtration, and integration of data [1]. Many databases are regularly updated, and the updated information is published in journals. Comprehensive information about the omics databases can be found by searching the Internet, including the website of the journal of nucleic acid research. On the next page in Table 1, we list a few of the important databases related to omics networks.

## 7. Conclusions

To understand the cell as a system, it is important to know the functions of different types of molecules at genome, transcriptome, proteome, and metabolome levels. At the same time, it is important to know how these molecules interact with each other and function as a whole. To achieve both these goals, the initial step is to construct their networks based on versatile biological information and to analyze such networks.

Some of the topological properties of a network such as degree distribution, average path length, and clustering coefficient can indicate which network model it belongs to among several network models, such as random, scale-free, and small-world models. Different centrality measures of the nodes can indicate important nodes in a network. Clustering of a biological network can determine biologically relevant groups of elements which can be utilized to extract novel biological information and predict the functions of some elements whose functions are not known.

In this review, we discussed the major molecular biological networks involving gene regulation, protein-protein interaction, metabolic, and signaling pathways. We also summarized the biological mechanisms and information relevant to such networks that are important for researchers working in the area of big data and network biology.

Omics networks have gradually become an indispensable part of biology and will become more and more useful in the future in various fields, including ecology and medicine. Despite their interrelations, signaling, protein-protein, gene regulatory, and metabolic networks frequently have been modeled independently in the context of well-defined subsystems. For such purposes, algorithms and mathematical formalisms have been developed according to the needs of each particular network under study. However, a deeper understanding of cellular behavior requires the integration of these various systems to discover how they cooperate with each other to function together.

## Figures and Tables

**Table 1:** A list of selected databases related to omics networks.

Database name	Short description	Web address
BioGRID [53]	A database of proteins, genes, and posttranslational modifications curated from the scientific literature	http://thebiogrid.org/

BRENDA [54]	A database of molecular and biochemical information on enzymes	http://www.brenda-enzymes.org/

ChEBI [55]	A database and ontology of molecular entities	http://www.ebi.ac.uk/chebi/

ChemSpider [56]	A database of chemicals. It contains more than 34 million unique molecules from over 450 data sources	http://www.chemspider.com/

DIP [57]	A manually curated database of experimentally elucidated interactions between proteins	http://dip.doe-mbi.ucla.edu/dip/

EcoCyc [58]	A database of the entire genome, and of transcriptional regulation, transporters, and metabolic pathways in *Escherichia coli* K-12, curated from the literature	http://ecocyc.org/

GenBank [59]	A comprehensive database of publicly available nucleotide sequences	http://www.ncbi.nlm.nih.gov/genbank/

Gene Ontology [60]	A database of annotations of genes and gene products for aiming to provide a common language to make data machine readable	http://geneontology.org/

IntAct [61]	A database of molecular interaction derived from literature curation or direct user submissions	http://www.ebi.ac.uk/intact/

InterPro [62]	A database for classification and analysis of protein sequences. It includes resources from PROSITE, HAMAP, Pfam, PRINTS, PRoDom, SMART, TIGRFAMs, PIRSF, SUPERFAMILY, CATH-Gene3D, and PANTHER	http://www.ebi.ac.uk/interpro/

KEGG [63]	An integrated database resource consisting of seventeen databases which are categorized into systems, genomic, chemical, and health information	http://www.genome.jp/kegg/

KNApSAcK [64, 65]	An integrated database constituting a database of manually curated metabolite-plant species, metabolic pathways, biological activities, and so forth for metabolomics studies	http://kanaya.naist.jp/KNApSAcK_Family/

MetaboLights [66]	A data repository for metabolomics experiments and obtained information	http://www.ebi.ac.uk/metabolights/

MetaCyc [67]	A metabolic database containing metabolic pathways, enzymes, metabolites, and reactions from many organisms	http://metacyc.org/

OMIM [68]	A database of diseases with human genes and genetic conditions	http://www.ncbi.nlm.nih.gov/sites/entrez?db=omim

ProteomeScout [69]	A database of proteins and post-translational modifications	https://proteomescout.wustl.edu/

PubChem [70]	A database of chemical molecules and their activities	http://pubchem.ncbi.nlm.nih.gov/

Reactome [71, 72]	A database of human biological processes authored by expert biologists	http://www.reactome.org/

RegulonDB [73]	A database of regulatory network and operon organization in *Escherichia coli* K-12	http://regulondb.ccg.unam.mx/

STRING [74]	A database of protein interactions (direct or indirect) derived from manual curation or text mining	http://string-db.org/

TAIR [75]	A comprehensive database of information and materials of *Arabidopsis thaliana*	https://www.arabidopsis.org/

TRANSFAC [76]	A manually curated database of eukaryotic transcription factors, their experimentally elucidated binding sites and DNA binding profiles. It needs a license for the up-to-date version	http://genexplain.com/transfac-db

TRANSPATH [77]	A database of mammalian signal transduction and metabolic pathways	http://genexplain.com/transpath-db

UniProtKB [78]	A database of functional information on proteins. It consists of UniProtKB/Swiss-Prot (manually annotated, reviewed) and UniProtKB/TrEMBL (automatically annotated, not reviewed)	http://www.uniprot.org/
